# Dynamic Contrast Enhanced-MR CEST Urography: An Emerging Tool in the Diagnosis and Management of Upper Urinary Tract Obstruction

**DOI:** 10.3390/tomography7010008

**Published:** 2021-03-02

**Authors:** Shaowei Bo, Farzad Sedaghat, KowsalyaDevi Pavuluri, Steven P. Rowe, Andrew Cohen, Max Kates, Michael T. McMahon

**Affiliations:** 1 The Russell H. Morgan Department of Radiology and Radiological Science, School of Medicine, The Johns Hopkins University, Baltimore, MD 21205, USA; boshaowei88@gmail.com (S.B.); fsedaghat@jhmi.edu (F.S.); phy.aim@gmail.com (K.P.); srowe8@jhmi.edu (S.P.R.); 2 The James Buchanan Brady Urological Institute, Department of Urology, School of Medicine, Johns Hopkins University, Baltimore, MD 21205, USA; acohen65@jhmi.edu (A.C.); Mkates@jhmi.edu (M.K.); 3 F.M. Kirby Research Center for Functional Brain Imaging, Kennedy Krieger Institute, Baltimore, MD 21205, USA

**Keywords:** urinary tract obstructions, ultrasound, CT, contrast enhanced MRI, CEST

## Abstract

Upper urinary tract obstructions (UTOs) are blockages that inhibit the flow of urine through its normal course, leading to impaired kidney function. Imaging plays a significant role in the initial diagnosis of UTO, with anatomic imaging (primarily ultrasound (US) and non-contrast computed tomography (CT)) serving as screening tools for the detection of the dilation of the urinary collecting systems (i.e., hydronephrosis). Whether hydronephrosis represents UTO or a non-obstructive process is determined by functional imaging (typically nuclear medicine renal scintigraphy). If these exams reveal evidence of UTO but no discernable source, multiphase contrast enhanced CT urography and/or dynamic contrast enhanced MR urography (DCE-MRU) may be performed to delineate a cause. These are often performed in conjunction with direct ureteroscopic evaluation. While contrast-enhanced CT currently predominates, it can induce renal injury due to contrast induced nephropathy (CIN), subject patients to ionizing radiation and is limited in quantifying renal function (traditionally assessed by renal scintigraphy) and establishing the extent to which hydronephrosis is due to functional obstruction. Traditional MRI is similarly limited in its ability to quantify function. DCE-MRU presents concerns regarding nephrogenic systemic fibrosis (NSF), although decreased with newer gadolinium-based contrast agents, and regarding cumulative gadolinium deposition in the basal ganglia. DCE-MR CEST urography is a promising alternative, employing new MRI contrast agents and imaging schemes and allowing for concurrent assessment of renal anatomy and functional parameters. In this review we highlight clinical challenges in the diagnosis and management of UTO, identify key advances in imaging agents and techniques for DCE-MR CEST urography and provide perspective on how this technique may evolve in clinical importance.

## 1. Introduction

**Figure 1:**
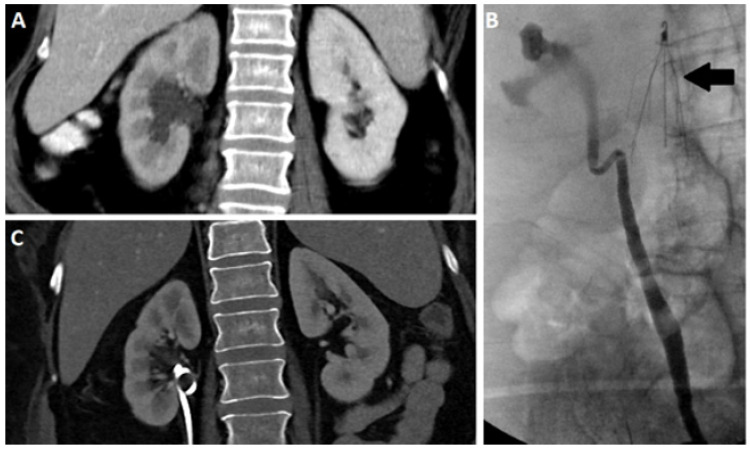
Coronal contrast enhanced CT (**A**) and retrograde pyelogram (**B**) depicting a hydronephrotic right kidney. Coronal CT after nephroureteral stent (**C**) placement demonstrating resolution of hydronephrosis. Note the presence of an IVC filter (arrow).

Upper urinary tract obstructions (UTOs) are blockages in the flow of urine that result in dilation of the renal collecting system (hydronephrosis), (Figure 1A,B), impaired glomerular/tubular function, renal injury, and, ultimately, irreversible loss of renal function. The most common cause of acute UTO in adults is nephrolithiasis, with patients presenting with unilateral flank pain secondary to an obstructing stone. Additional causes of upper UTOs vary according to age and sex, urothelial malignancy, benign strictures and congenital ureteropelvic junction (UPJ) obstruction. While an elevation in creatine and decrease in eGFR may be observed, fulminant renal failure is rare in the setting of unilateral UTO and the absence of pre-existing chronic kidney disease (CKD). These entities often have an insidious presentation and may go undetected, leading to irreversible kidney damage. Thus, the early identification of functional renal impairment is vital in prompting intervention and the preservation of renal function.

The treatment of upper UTOs depends on the cause of the obstruction and degree of renal impairment. For example, in the setting of nephrolithiasis, conservative management is often the choice for small stones, as these spontaneously pass and rarely cause permanent functional impairment. Conversely, lithotripsy and stenting are the standard treatment for larger obstructing calculi. In other settings where hydronephrosis is present without significant functional impairment, treatment centers around addressing the underlying etiology for UTO. UTO related to malignancy is typically managed surgically, irrespective of the degree of functional impairment. However, in some cases, the quantification may still play a role, particularly in determining the utility of intervening to preserve a hydronephrotic kidney. When functional impairment/acute kidney injury (AKI) is secondary to partial or complete ureteral obstruction, percutaneous nephrostomy (Figure 1C) and retrograde ureteric stenting are well-established choices for minimizing renal parenchymal injury and preserving renal function. While these treatments are generally safe and well-tolerated, they are not without risk and there is inherent morbidity related to the presence of urinary stents, which can become a nidus of infection. Furthermore, the presence of hydronephrosis, and even the documentation of a functional UTO, does not suggest whether damage from that UTO is reversible. Interventions are, therefore, undertaken to alleviate obstructions without knowing if the intervention will actually improve kidney function. Finally, monitoring UTO after surgical intervention is a diagnostic conundrum. Interventions, such as ureteric surgeries or even ureteric stents, are often associated with persistent hydronephrosis. In the case of stents, such persistent hydronephrosis may or may not suggest incomplete treatment and persistent obstruction. For UPJ obstruction, evidence of obstruction often persists on imaging even after a treatment is deemed successful by other subjective and objective means. Such patients are at high risk for functional impairment given the prior trauma to their renal unit, but detection is confounded by the abnormal appearance of their kidneys. Ultimately, patients with residual hydronephrosis after surgical intervention often undergo multiple repeated testing with varied modalities in an effort to catch further renal deterioration early in its course. Due to limitations to current imaging modalities, these efforts have unclear clinical efficacy [1,2].

Standard blood and urine-based laboratory tests, while vital to the assessment of renal function, are somewhat limited in defining the reversibility of functional impairment related to upper UTO. Estimations of the glomerular filtration rate (eGFR) derived from serum creatinine measurements, as well as demographic and anthropomorphic information, can determine how well the kidneys remove waste and excess fluid from the blood, and are a highly cost-effective assessment of kidney function. Generally, an eGFR < 60 suggests renal impairment. Lower eGFR estimates may indicate worsening degrees of renal failure, with eGFR < 15 indicating fulminant renal failure, requiring either dialysis or transplantation. While these eGFR estimates are helpful in screening patients for renal impairment, they indicate little about the chronicity of renal injury and the reversibility of renal impairment in the setting of UTO. If prior eGFR measurements are available, values can be trended, and if a precipitous drop in eGFR accompanies the development of UTO/hydronephrosis, one can deduce a causal relationship. Generally, if the impairment is relatively new, it can be surmised that the renal impairment is largely reversible. However, in practice prior imaging and laboratory tests are often unavailable, and confounding factors ranging from medical illness, race [3], to hydration status may skew eGFR results [4]. Thus, in most instances the decision to intervene on UTO is often contingent on clinical judgment rather than objective criteria.

## 2. Imaging Modalities

**Figure 2:**
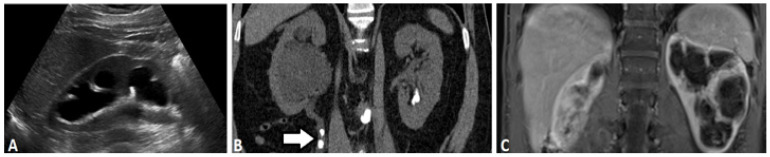
Different imaging modalities of kidney including right renal ultrasound (**A**), coronal non-contrast CT (**B**) and contrast enhanced MRI (**C**) demonstrating unilateral hydronephrosis. Note the presence of obstructing proximal uretal stones in the CT (arrow).

GFR and renal perfusion can be used for diagnosis of kidney diseases and monitoring of treatment interventions. Measuring the clearance of exogenous markers in patient’s blood is considered to be the gold standard for GFR measurement. A number of imaging options are available for patients with suspected UTOs, including ultrasound, X-ray (including intravenous urography (IVU)), computed tomography (CT), nuclear medicine renography, and magnetic resonance imaging (MRI) (Figure 2). We review the current clinically available options first.

### 2.1. Ultrasound-Based Urography

Renal ultrasound, which is fast, noninvasive, and easily accessible, is widely used in the initial evaluation of the urinary system (Figure 2A). Renal ultrasound can be used to assess the size, shape, and location of the kidneys, and look for the presence of hydronephrosis, a sensitive-but-not-specific sign of UTO. As with other anatomic imaging modalities, renal ultrasound can identify the presence of hydronephrosis but cannot generally determine if that hydronephrosis is due to a UTO or a non-obstructive pathology. Increases in renal cortical echogenicity, assessed relative to the liver if the right kidney is in situ, are generally indicative of underlying medial renal disease. Furthermore, in most instances, renal masses and stones are also evident. Nonetheless, definitively identifying the etiology of a UTO based on ultrasound is often challenging because of several inherent limitations [5,6]: (1) acquisition and interpretation of ultrasound images depends on the operator; (2) renal ultrasound cannot visualize the full length of the ureter unless it is significantly dilated; (3) the resolution and tissue penetration depth are not sufficient to detect smaller pathological defects. Even with these drawbacks, ultrasound is still used as a valuable first-line investigation to rule out obstructive nephropathy in patients presenting with laboratory evidence of acute kidney injury (AKI) [7] and/or suggestive symptoms. Additionally, contrast-enhanced ultrasound (CEUS) using microbubbles is an emerging technique increasingly employed for imaging of the kidneys. At this stage, CEUS is primarily used for the evaluation of renal masses, although it has also shown promise in assessing renal perfusion.

### 2.2. X-ray Based Urography

Intravenous and retrograde urography have been mainstays for renal imaging prior to the advent of cross-sectional imaging. In X-ray urography the urinary system is opacified by iodinated contrast, which is administered intravenously and excreted or retrograde via foley catheter [8]. While reliably detecting hydronephrosis (Figure 1B), X-ray urography provides limited sensitivity in detecting many important urological pathologies, most notably small urothelial lesions and renal masses. Furthermore, while IVU may demonstrate delayed excretion, indicating functional impairment, improved diagnostic accuracy can be achieved with newer imaging methods, including CT and MRI.

### 2.3. Computed Tomography

Computed Tomography (CT), which utilizes a helical array of X-rays to produce volumetric tomographic images, has largely supplanted IVU in the assessment of UTOs (Figure 2B). CT provides excellent spatial and contrast resolution and utilizes iodinated agent contrast, effectively delineating the kidneys, collecting systems, ureters, and bladder [9,10,11,12]. Unenhanced CT, while lacking the contrast resolution of a multi-phasic contrast enhanced exam, is nonetheless effective in identifying hydronephrosis, and is the standard bearer for detection of radiodense stones. The presence of hydronephrosis, without a discernable cause, is often further assessed with multi-phasic CT. By obtaining non-contrast, corticomedullary (approximately 30 s), nephrographic (60 s), and delayed phases (5–8 min), one can identify otherwise occult causes of UTO, most notably renal/urothelial tumors. Furthermore, the persistence of nephrographic appearance in the hydronephrotic kidney and absent or reduced contrast excretion indicates accompanying functional impairment, although again the degree of impairment cannot be reliably quantified. Further, hydronephrosis on CT can indicate either UTO or non-obstructive pathology, and in many cases the distinction cannot be determined based only on imaging findings. Radiation exposure is an important limitation of CT, as well as contrast-induced nephropathy (CIN) by iodine contrast media, which present risks for patients with impaired kidney function. Despite these limitations, CT remains a mainstay of renal imaging.

### 2.4. Nuclear Scintigraphy

Nuclear scintigraphy is currently the gold standard for the assessment of differential kidney function and for evaluating UTOs. The fundamental reason for the predominance of nuclear medicine scintigraphy in evaluating UTOs is the ability to differentiate a patulous renal collecting system from an obstructive uropathy [13]. This approach is based on time resolved planar imaging of the kidneys after intravenous administration of a radiotracer that is either filtered (^99m^Tc-diethylene triamine pentacetic acid, ^99m^Tc-DTPA) or secreted by the tubules (^99m^Tc-mercapto acetyl triglycine, ^99m^Tc-MAG3). As such, nuclear medicine scintigraphy is fundamentally based on physiology and not anatomy. In cases in which a patient has evidence of hydronephrosis from anatomic imaging, the patient should undergo nuclear medicine scintigraphy in order to determine whether the hydronephrosis is due to a UTO or a result of non-obstructive pathology. In fact, ^99m^Tc-MAG3 is particularly useful due to its mechanism of clearance being tubular secretion-based; as a result, it is usable even with depressed eGFR due to renal dysfunction [13].

**Figure 3:**
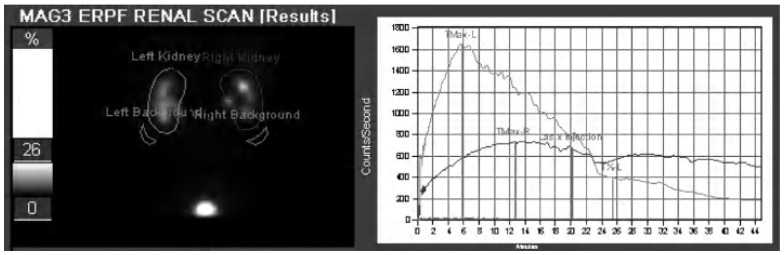
MAG-3 renogram and time activity curve of the patient with obstructing ureteral calculi depicted in Figure 2B. Note the marked asymmetrically decreased right renal counts and decreased and prolonged clearance, confirming the presence of obstructive uropathy and indicating significant functional impairment.

Scintigraphic evaluation can be enhanced by use of Lasix as a diuretic. In situations in which the clearance of radiotracer from the renal collecting systems is slow or absent, Lasix can be administered in order to increase the production of urine and overcome any intrinsic patulousness in the system. If a hydronephrotic collecting system clears rapidly after administration of Lasix, the system is dilated but not obstructed. If, on the other hand, the system clears slowly or not at all (Figure 3), the findings are indicative of functional obstruction. This use of physiology to discern the nature of hydronephrosis is unique among currently available imaging techniques. However, this technique suffers from low spatial resolution and does not provide detailed anatomic information; therefore, a cause of UTO may not be appreciated. Renal scintigraphy is sensitive to patient details and does involve ionizing radiation—therefore, it can have limitations in use in pediatrics [13].

### 2.5. Magnetic Resonance (MR) Based Urography

**Figure 4:**
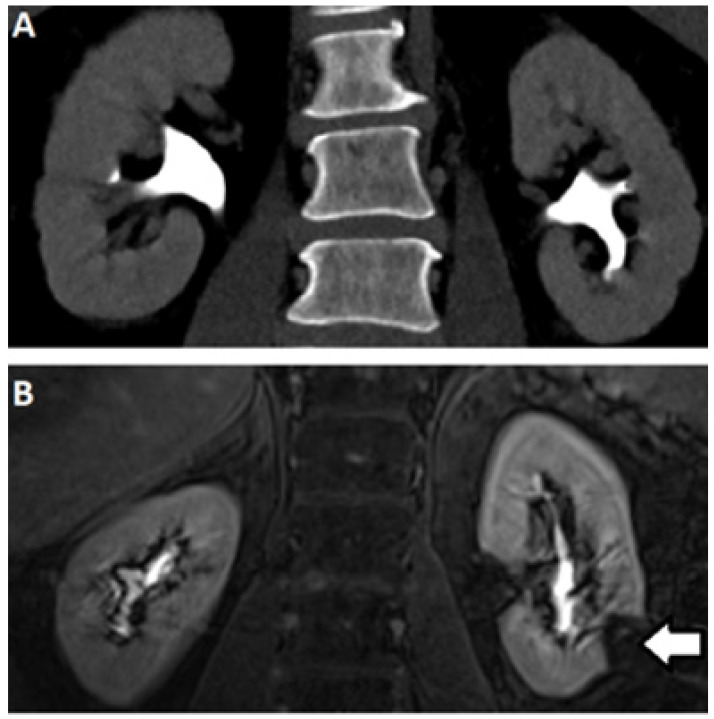
Excretory phase CT (**A**) and MRI (**B**) demonstrating normal caliber renal collecting systems without filling defect. Note the absence of signal in the left lower pole (arrow) corresponding to a renal cyst.

MR urography is an alternative technique for the detection of urothelial lesions and strictures. MRI utilizes magnetic fields and radiofrequency pulses without imparting ionizing radiation and displays soft tissue contrast due to differences in water density and relaxation. Two MR urography methods are currently employed: static-fluid MR urography and excretory Dynamic Contrast Enhanced MR urography (DCE-MRU). Static-fluid MR urography exploits the long transverse relaxation times (T_2_) of urine, with heavily-fluid weighted sequences that suppress short T_2_ proton signals with long echo times, typically acquired using single-shot fast spin-echo (SS-FSE) and half-Fourier single-shot spin echo (HASTE) sequences to provide outstanding visualization of the urinary tract. This is of particular concern in UTO, where an obstructed kidney may generate little urine. Furthermore, dynamic cinematic acquisitions can be obtained, allowing areas of ureteral peristalsis to be distinguished from fixed stenoses. Excretory DCE-MRU urography currently relies on excreted MRI imaging agents (mainly based on gadolinium) to generate signal changes within the collecting system (fat suppressed T_1_ acquisition, typically obtained using gradient-echo techniques). Both MRU approaches avoid use of ionizing radiation and pose no risk of CIN, which are advantages over CT. They have none of the technical limitations of US. Static fluid sequences do not require administering gadolinium contrast, avoiding concerns related to NSF and cumulative gadolinium deposition. As shown in Figure 2C and Figure 4, the resulting images possess better soft tissue contrast compared to other modalities. In practice, static and DCE-MRU are often employed in tandem with accompanying multiphasic imaging of the kidneys and/or bladder.

## 3. The Emergence of New DCE-MRU Technologies

A number of new strategies have been identified for using DCE-MRU to measure renal perfusion [14,15,16,17,18,19,20,21]. DCE-MRI is contingent on renal function, and similar to CT, it can reveal renal functional impairment, although unlike CT it is possible to acquire many time points to more accurately quantify functional impairment. Currently, gadolinium-based contrast agents (GBCA) are the most widely used agents which rely on reducing the T1 of neighboring water molecules to impart signal changes [22,23,24,25]. Rapid multi-slice methods to extract contrast kinetics have been developed for the comprehensive assessment of kidney function [19,23,24,26,27,28]. Administering GBCAs can provide functional characterizations of obstructions [29,30,31] and for moderately dilated renal collecting systems, the MRI measurement of split renal function was proven to be equivalent to renal scintigraphy whereas for severely dilated kidneys there was an underestimate of split renal function [32]. Another retrospective study with a larger set of patients found limitations in the precision of GBCA determinations of split renal function [33]. To offset these limitations found using GBCAs, alternative imaging agents can be applied including: ^19^F imaging agents [34], hyperpolarized ^129^Xe imaging agents [35], hyperpolarized ^13^C spectroscopic imaging agents [36] and CEST contrast agents [37]. Fain and colleagues demonstrated the power of hyperpolarized ^13^C pyruvate for the evaluation of partial ureteral obstruction in mouse models, showing that differences in pyruvate metabolism for obstructed and unobstructed kidneys can be seen using this imaging agent [36]. However, chemical exchange saturation transfer (CEST) contrast agents represent a particularly promising technology due to their capabilities of detect changes in metabolism, perfusion and pH [38] which we will discuss further below. With all these MR imaging agents displaying promise in preclinical studies, DCE-MRI of the kidneys is a vibrant and active area of investigation.

### 3.1. DCE-MR CEST Urography

CEST has emerged as an MRI contrast mechanism that can be used to detect small amounts of contrast agent through an amplified detection of low concentration protons (on the order of low mM) through their exchange with water [38]. Based on the early work of Cerdan, Gillies, Sherry, Bhujwalla and colleagues, pH imaging is an emerging field [39,40,41,42,43,44,45,46]. This includes the outstanding work of Andreev, Reshetnyak, Lewis, Gao and colleagues developing both fluorescent and PET probes to detect low pH environments [47,48,49,50,51]. CEST MRI is now one of the premier imaging methods for the measurement of pH due to proton chemical exchange being sensitive to acid-base equilibrium, the development of ratiometric methods for distinguishing changes in agent concentration from changes in pH, and the amplified detection of these agents compared to spectroscopy [52,53,54,55,56,57,58,59,60,61,62,63,64,65,66,67]. Traditional CT and MR urography, while effective at characterizing upper tract function, cannot reliably quantify renal function, often requiring subsequent renal scintigraphy exams. Conversely, CEST MRI can characterize urinary tract obstruction and function simultaneously, generating pH maps in addition to traditional time activity curves seen on renal scintigraphy. The novel ability of CEST imaging to measure pH is of particular import, as renal functional impairments are often associated with a urinary acidification defect caused by diminished net H^+^ secretion and/or HCO_3_^−^ reabsorption. While this can be assessed systemically through measurement of urine pH, using MR to image tubular pH can best assess whether a patient is experiencing functional decline in their obstructed renal unit, quantify the amount of renal functional impairment, and even assess whether this functional impairment is reversible (thus potentially benefiting from intervention) or irreversible. Additionally, in the case of urolithiasis, pH is relevant due to its relationship with stone formation, with urine acidification or alkalization employed as treatments depending on the stones’ constitution. Hence, CEST imaging is of interest in the evaluations of upper UTOs not only due to efficiency, but the ability to provide novel functional information to inform clinical therapy.

**Figure 5:**
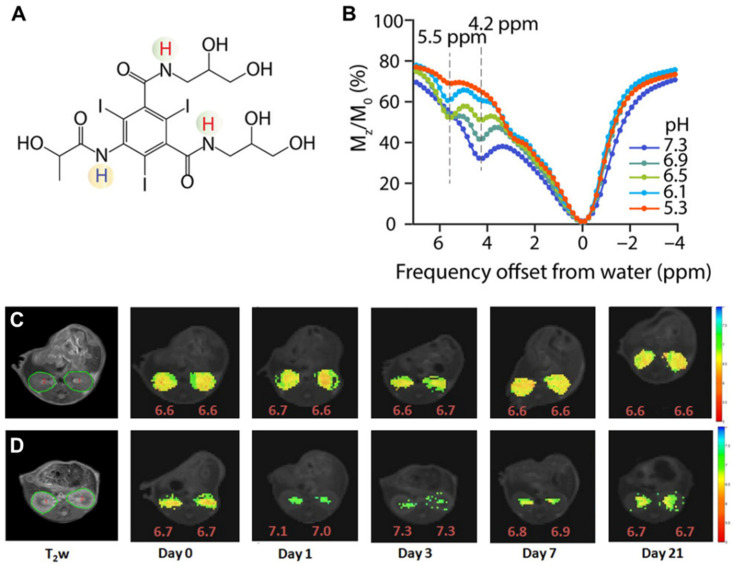
Iopamidol as pH imaging agent tested in mice. (**A**) Structure of iopamidol with exchangeable protons highlighted, which produce CEST contrast at 4.2 and 5.5 ppm; (**B**) CEST Z-spectra of iopamidol in blood serum for pH = 5.3, 6.1, 6.5, 6.9, and 7.3. Panels (**C**,**D**) adopted from Pavuluri et al. in [70]; Representative anatomical (T_2w_) and superimposed color-coded pH maps obtained 15 min after Iopamidol injection at indicated time points in control group (**C**) and in AKI group (**D**). Panels (**C**,**D**) adopted from Longo et al. in [69].

**Figure 6:**
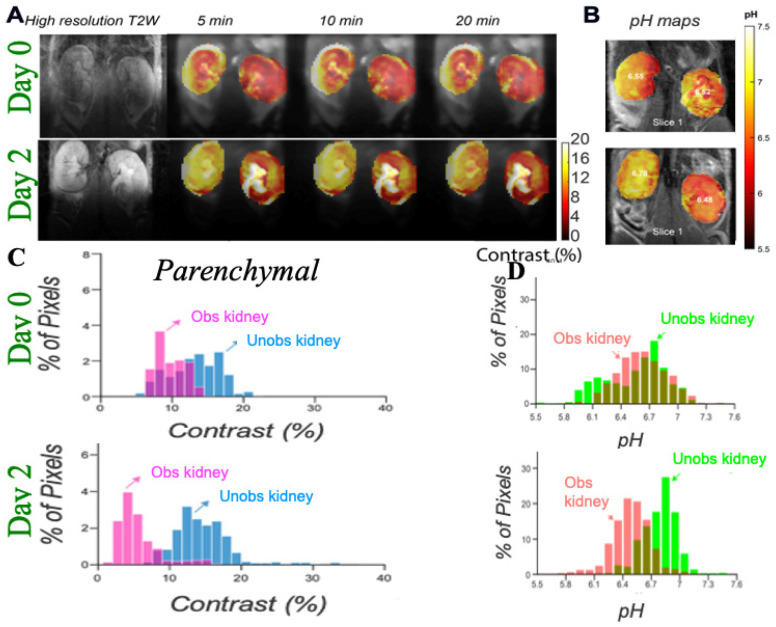
Imaging UUO in mice using iopamidol CEST MRI; (**A**) Representative T2w and CEST MRI contrast maps at 4.2 ppm on a UUO mouse with right kidney obstructed on day 0 and 2 post surgery with iopamidol dose = 1 g I/kg, B_1_ =4 μT, 2 offset protocol, as in reference [70], to minimize pH mapping time and allow averaging for the production of high contrast to noise ratio (CNR) motion artifact compensated CEST maps; Dynamic CEST images were acquired at offsets 4.2, 5.5 ppm repeatedly for 80 min using a CW RF saturation of duration 2.1 s (7 rectangular block pulses each of 300 ms with 10 μs interpulse delay). TE/TR = 3.55/11,000 ms; Rapid Acquisition with Relaxation Enhancement with short echo time (RAREst) acquisition module and centric encoding were used. In total, 10 sets of M0 offsets at 40 ppm were acquired for normalization. Time interval between two dynamic CEST images was 44 s. Moving time average of 10 dynamic CEST images was performed to compensate the motion induced artifacts in CEST contrast with the B1 employed rendering the maps sufficiently insensitive to the B0 homogeneity across the kidneys shown in the B0 maps as described in reference [70]. The images depict differences in marker perfusion for left and right kidneys due to hydronephrosis and resulting functional impairment; (**B**) pH maps using images acquired 5 min after iopamidol administration. pH was calculated using the ratio of CEST contrast at 4.2, 5.5 ppm and the calibration curve obtained using the iopamidol phantom at pH values between 5.3 and 7.3; (**C**) Parenchymal contrast histograms which display reduced contrast for obstructed kidney cortex and a larger variation in contrast for the obstructed kidney calyx; (**D**) pH histograms which display an increase in ΔpH over time after UUO first in obstructed kidney and later in unobstructed kidney which is similar to what was observed in MMA induced CKD in mice.

The triiodobenzenes iopamidol (ISOVUE^®^, Figure 5A) and iopromide were the first agents with particular promise identified for CEST imaging of the kidneys [66,68]. These are safe, nonionic molecules that have been in clinical use for over 30 years as X-ray contrast agents and administered at very high doses (up to 400 mg/mL). They both also have two exchangeable amide proton resonances (for iopamidol 4.2 ppm and at 5.5 ppm downfield from the water signal, Figure 5B) that produce pH-dependent CEST contrast. Ratiometric methods for pH assessment have been developed based on comparison of the signals at the frequencies of these two amide proton resonances to measure pH within the range of 5.5 to 7.4. Longo and colleagues evaluated if iopamidol-based CEST MR could be used to detect the recovery of kidney function in an ischemia reperfusion acute kidney injury (AKI) rodent model using two metrics: renal perfusion and renal pH [69]. A return to normal perfusion and pH values was observed by Day 7 for moderate lengths of ischemia induction, whereas a persistent drop in the perfusion of contrast agent and increase in renal pH was observed for longer induction times (Figure 5C,D). Our group evaluated if iopamidol-based CEST MR could be used to detect progression in chronic kidney disease (CKD) in a the methyl malonic acidemia (MMA) model [70]. This was investigated using four groups of mice: healthy controls on a regular diet (RG *Mu^+/−^*) and high protein diet (HP *Mu^+/−^*), mild kidney disease mice kept on a regular diet (RG *Mu^−/−^*), and severe kidney disease mice on a high protein diet (HP *Mu^−/−^*). Both RD and HP *Mu^+/−^* controls displayed homogeneous pH values of 6.5 and excellent kidney perfusion of agent (~100%) while, in contrast, RD *Mu^−/−^* mice displayed a lower average pH (~6.1) and perfusion (~79%) and an order of magnitude larger range of pH values in the kidneys (±0.2). Furthermore, HP *Mu^−/−^* mice displayed a slightly lower pH (~6.0), substantially lower perfusion (~15%) and significantly larger range of pH values (±0.45). We have further tested iopamidol-based CEST MR on a complete unilateral urinary obstruction (UUO) mouse model. As shown in Figure 6, there are, again, changes in both iopamidol perfusion, average pH and range in pH values could be visualized. Other important work has been performed to improve the imaging protocols and establish these agents on different models of kidney disease and for tumor imaging as well [37,56,71,72]. The results have demonstrated that iopamidol-based CEST MRI pH mapping is promising for monitoring of renal function, allowing for an early detection of the occurrence of renal pathology and distinguishing moderate from severe AKI.

**Figure 7:**
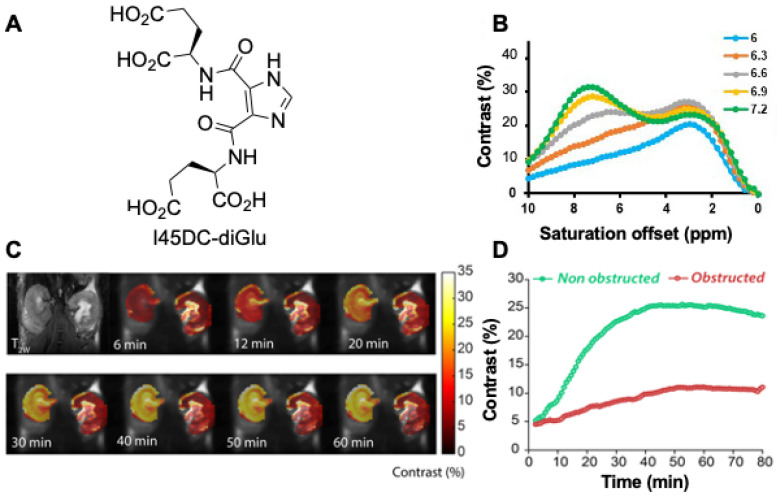
Imaging unilateral urinary obstruction in mice using Imidazole CEST MRI agent; (**A**) Imidazole-4,5-dicarboxamide-di Glutamate (I45DC-diGlu) structure; (**B**) Experimental MTR_asym_ spectra for I45DC-diGlu as a function of pH t_sat_ = 3 s, ω_1_ = 6.0 μT, 37 °C; (**C**) in vivo kidney images; Experimental conditions: t_sat_ = 3 s, ω_1_ = 6.0 μT. Dynamic CEST images were acquired using the same 2 offset CEST protocol described in Figure 6 for Iopamidol. Two CEST images at offsets 4.3 and 7.5 ppm were acquired repeatedly for a total time of 76 min and 10 set of M0 images at offset 40 ppm were collected. Time interval between two dynamic CEST images was 44 s; (**D**) Average parenchymal contrast for obstructed and non-obstructed kidney.

Other agents have also shown promise for kidney imaging. For example, Sherry and colleagues performed in vivo pH imaging of mouse kidneys using a frequency-dependent lanthanide-based CEST agent [73]. Other agents tested include urea and phosphocreatine [74,75]. Our group has synthesized imidazole CEST imaging agents including Imidazole-4,5-dicarboxamide-di Glutamate (I45DC-diGlu) [76], and, as is shown in Figure 7, seems particularly promising for evaluating UTOs. This agent is well suited for ratiometric pH imaging on 3 Tesla scanners with two labile protons with large chemical shifts that produce strong pH sensitive contrast (Figure 7A,B). Figure 7C,D display higher kidney contrast and a larger difference in split renal contrast than seen using iopamidol. A number of well-tolerated medications have been developed based around imidazoles [77], including for treatment of cancer [78], ulcers [79,80], hypertension [81] and as antihistaminic drugs [82] which is encouraging for translating imidazole MRI contrast agents. We expect that one of the CEST agents mentioned above, or perhaps one not yet discovered, will prove outstanding for functional DCE-MR CEST urography of UTOs.

### 3.2. CEST MRI at 3T Using Iopamidol

**Figure 8:**
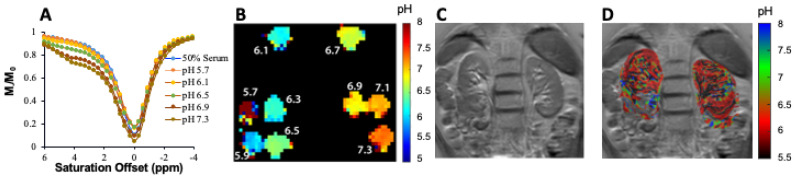
Example of ratiometric measurement of pH using 25 mM iopamidol in blood serum at 3 T using B_1_ = 2 μT, (**A**) CEST z-spectra at pH = 5.7, 6.1, 6.5, 6.9 and 7.3 acquired for 63 offsets between −4 and 6 ppm; (**B**) pH maps of phantom; (**C**) T2 image of the abdomen in first healthy subject; (**D**) pH map of the kidneys after injection of iopamidol. CEST data were acquired for 18.9 min using t_sat_ = 2 s, ω_1_ = 1.5 μT and TR = 6 s, at repeated offsets = 20,000, 6.1, 5.6, 5.1, 4.6 and 4.1 ppm, respectively. CEST contrast at 4.6 and 5.6 ppm was used for pH calculation. This set of offsets was necessary to partially compensate for the B0 homogeneity shown in the B0 maps across the kidneys on this scanner.

At this stage, all of the major clinical scanner vendors have sequences for performing CEST imaging, allowing for the translation of pH imaging to patients [83,84,85,86,87]. Based on the success of our studies in mice, we prepared an iopamidol phantom at several pH values in serum and moved to establishing a CEST imaging protocol on our 3T Philips Achieva scanner (Philips Healthcare Solutions, Amsterdam, NL, USA) to test how well we could create pH maps. Example data are shown in Figure 8. As is shown, pH mapping could be performed over the range of 5.9 to 7.3 on our scanner; however, below this pH the performance was not ideal. Based on this, we injected iopamidol into our first healthy subject (Isovue 300, 90 mL injection volume) and observed ~4% contrast across both kidneys which was strong enough to enable generation of our first pH maps on a human subject (Figure 8D). Improvements in imaging protocol and post processing are currently being implemented and are expected to yield higher contrast to noise ratio pH maps. These results indicated that DCE-MR CEST urography is very promising for translation into patients with UTOs.

## 4. Conclusions

Early diagnosis and treatment of UTOs can salvage kidney function. CE-MRI methods have shown great potential for the evaluation of kidney function, especially CEST MRI, which can evaluate renal perfusion, quantify contrast enhancement, and effect pH mapping, which can provide more detailed information for the kidneys. In the case of upper UTOs the hope is that these new technologies might enable earlier detection of whether there is functional renal impairment and if interventions will preserve renal function. CEST MRI also shows the potential to evaluate kidney function. If CEST MRI contrast agents could provide more information that relates to renal functional impairment, DCE-MR CEST urography might become more mainstream for the assessment of upper UTOs [24] and be used in the clinic for assisting in decision making regarding treatment for patients with kidney diseases.
